# Predictive survival modelings for HIV-related cryptococcosis: comparing machine learning approaches

**DOI:** 10.3389/fcimb.2025.1542707

**Published:** 2025-05-02

**Authors:** Xuemin Fu, Luling Wu, Jingna Xun, Benno Pütz, Zhihang Zheng, Yanpeng Li, Yinzhong Shen, Hongzhou Lu, Jun Chen, Bertram Müller-Myhsok

**Affiliations:** ^1^ Statistical Genetics, Max Planck Institute of Psychiatry, Munich, Germany; ^2^ Department of Infectious Diseases and Immunology, Shanghai Public Health Clinical Center, Fudan University, Shanghai, China; ^3^ Department of Endoscopy, Shanghai Pulmonary Hospital, School of Medicine, Tongji University, Shanghai, China; ^4^ National Clinical Research Center for Infectious Disease, Shenzhen Third Peoples Hospital, Second Hospital Affiliated to Southern University of Science and Technology, Shenzhen, China; ^5^ Shanghai Public Health Clinical Center, Fudan University, Shanghai, China; ^6^ Hubei Jiangxia Laboratory, Wuhan, China

**Keywords:** HIV, cryptococcosis, machine learning, survival prediction, cytokine, penalized Cox regression

## Abstract

**Introduction:**

HIV-associated cryptococcosis is marked by unpredictable disease trajectories and persistently high mortality rates worldwide. Although improved risk stratification and tailored clinical management are urgently needed to enhance patient survival, such strategies remain limited.

**Methods:**

We analyzed clinical and immunological data from 98 HIV-related cryptococcosis cases, employing machine learning techniques to model disease severity and predict survival outcomes. Our approach included unsupervised clustering, elastic net regularized Cox regression, and random survival forests. Model performance was rigorously assessed using the C-index, Brier score, Calibration and time-dependent AUC, with validation executed through a comprehensive, multi-replicated nested cross-validation framework.

**Results:**

Through cytokine profiling, we identified an immune phenotype characterized by excessive inflammatory response (EXC), associated with greater disease severity, more frequent neurological symptoms, and poorer survival outcomes compared to the other two immune phenotypes, highlighting its potential significance in risk stratification. To further support clinical decision-making, we developed an elastic net regularized Cox regression model, achieving superior predictive accuracy with a mean C-index of 0.78 for 36-month outcomes and a mean Brier score of 0.13, outperforming both random survival forest and traditional Cox models. Time-dependent AUC analysis validated the model’s robustness, with AUC values of 0.84 at 12 months and 0.79 at 36 months, indicating its reliability and potential clinical utility.

**Discussion:**

This study presents comprehensive and multidimensional approaches to overcome the challenges commonly encountered in real-world clinical settings. By applying cytokine-based clustering, we illustrate the potential for more nuanced severity stratification, offering a fresh perspective on disease progression. In parallel, our penalized survival model provides a step forward in personalized risk assessment, supporting informed clinical decisions and customized patient management. These findings suggest promising directions for individualized healthcare solutions, leveraging machine learning to enhance survival predictions in HIV-related cryptococcosis.

## 1 Introduction

HIV-associated cryptococcosis remains a severe, life-threatening opportunistic infection, especially among individuals with profound immunosuppression (Pasquier et al., 2018). Despite advancements in antiretroviral therapy (ART), cryptococcal meningitis continues to pose high morbidity and mortality rates worldwide, particularly in low- and middle-income countries (Pasquier et al., 2018). The rapid and unpredictable progression of the disease underscores the urgent need for effective risk assessment tools to identify high-risk patients early and enable timely, targeted interventions to improve clinical outcomes.

While numerous models have been developed to predict HIV infection risk, guideline-endorsed tools for widespread clinical use remain lacking due to challenges in generalizability, data completeness, and practicality for clinical integration (Li et al., 2024). Additionally, Cytokine dysregulation and inflammation likely contribute to CD4^+^ T cell depletion and persistent viral load in HIV pathogenesis (Hunt PW., 2012). More studies were incorporating diverse data sources into clinical research, to our knowledge, limited research has involved multiplex cytokine profiling in predictive models.

The complexity of HIV, with its various complications, poses significant challenges for traditional statistical models like Cox proportional hazards (Cox PH) for survival analysis (Cox, 1972) and logistic regression for non-survival outcomes (Efthimiou et al., 2024). While valued for their interpretability, these models face limitations of multicollinearity and non-linear effects when handling multiple features. In contrast, machine learning (ML) models excel at managing large, complex datasets by automatically identifying intricate patterns without strict statistical assumptions (Waring et al., 2020). They can integrate diverse data types—such as immunological markers and clinical parameters—for more accurate predictive models. Commonly used ML models in clinical research include support vector machines (SVM) and random forests (RF), both demonstrating strong predictive performance (Liu et al., 2020). However, for survival analysis, random survival forests (RSF) are among the few ML-based approaches gaining traction, as traditional Cox regression remains predominant despite the advantages offered by more flexible ML techniques (Li et al., 2024).

Large datasets are typically preferred for developing robust models and ensuring accurate evaluations. However, real-world clinical trials—particularly those addressing HIV-associated conditions—often struggle with limited sample sizes. Relying on simple train-test splits can yield biased performance estimates and poor generalizability due to variability in data division. To mitigate these risks, techniques such as cross-validation (CV) and leave-one-out cross-validation (LOOCV) are commonly used (Vabalas et al., 2019). More recently, nested cross-validation(nested CV) has emerged as a superior approach, utilizing an inner loop for model tuning and an outer loop for performance evaluation (Vabalas et al., 2019). This method maximizes data efficiency, provides more reliable performance metrics, and minimizes the risk of overfitting, which is crucial when dealing with small datasets and complex clinical variables.

To tackle these challenges, our study seeks to develop and validate reliable survival prediction models by employing advanced machine learning techniques and combining immunological and clinical data, specifically for HIV-associated cryptococcosis. We utilize nested cross-validation to ensure our findings are robust and generalizable, ultimately contributing to more accurate and practical tools for clinical decision-making—even in resource-limited settings.

## 2 Materials and methods

### 2.1 Data collection

We collected data from all patients with HIV-associated cryptococcosis admitted to the Shanghai Public Health Clinical Center between January 1, 2016, and June 1, 2024. After filtering for completeness of demographic data, clinical features, laboratory tests, and CT imaging characteristics, a total of 98 patients with high-quality plasma samples were included in this study. Disease severity for all patients was assessed within 24 hours of admission using the Sequential Organ Failure Assessment (SOFA) score, the Confusion, Urea nitrogen, Respiratory rate, Blood pressure, and Age ≥65 years (CURB-65) severity score, the Acute Physiology and Chronic Health Evaluation (APACHE-II) scoring system, the Veterans Aging Cohort Study (VACS) 2.0 index score, and the Glasgow Coma Scale (GCS) (McGinnis et al., 2022; Rosas-Carrasco et al., 2022). All patients received antifungal therapy upon diagnosis of cryptococcosis, with antiretroviral therapy (ART) initiated at least 4–6 weeks after the start of antifungal treatment (Chang et al., 2024). Plasma samples were collected prior to any treatment and sent to the specimen bank of the Infection and Immunology Department at the Shanghai Public Health Clinical Center. Samples were centrifuged at 1000 g for 20 minutes, divided into aliquots, and stored at –80° C until further experimentation. All samples underwent a quality screening process before subsequent testing.

### 2.2 Experimental measures and preprocessing

#### 2.2.1 Cytokines and chemokines

A total of 98 plasma samples were experimented with the multiplex ELISA method (Bio-Plex Pro Human Cytokine 27-plex Assay, catalog no: #M500KCAF0Y, Bio-Rad Laboratories, Inc., Hercules, CA, USA). The following cytokines and chemokines were measured: interleukins (IL)-1β, IL-2, IL-4, IL-5, IL-6, IL-7, IL-8, IL-9, IL-10, IL-12p70, IL-13, IL-15, IL-17, IFNγ, tumor necrosis factor-α (TNFα), interferon-inducible protein-10 (IP-10), IL-1RA, monocyte chemoattractant protein-1 (MCP-1), macrophage inflammatory protein-1α (MIP-1α), macrophage inflammatory protein-1β (MIP-1β), platelet-derived growth factor-BB (PDGF-BB), RANTES, granulocyte–macrophage colony-stimulating factor (GM-CSF), granulocyte colony-stimulating factor (G-CSF), vasoactive endothelial growth factor (VEGF), fibroblast growth factor (FGF), and Eotaxin. Cytokine and chemokine levels were quantified using Bio-Plex Manager software. Values below the detection threshold were recorded as zero, indicating undetectable levels. Values exceeding the upper limit of the standard curve were considered out of the assay’s scope and assigned the maximum value on the curve.

#### 2.2.2 Flow cytometry assays

Peripheral blood mononuclear cells (PBMCs) from prior-treatment patients were isolated by Ficoll-Paque density gradient centrifugation and seeded in 96-well plates (2 × 105 cells/well). Cells were stimulated with purified cryptococcal mannoprotein for cryptococcal antigen (CrAg) detection. The cryptococcal mannoprotein-free stimulation group served as an autologous control, while PMA/ionomycin-treated cells (eBioscience, USA) served as positive controls. Following 2-hour incubation, protein transport inhibitor (eBioscience, USA) was added, and cells were cultured for an additional 8 hours.

Cell viability was assessed using Live/Dead Fixable Violet dye (Invitrogen, USA). Surface markers were analyzed using anti-human CD3-Alexa Fluor 700 (Clone OKT3; BioLegend, USA), anti-human CD4-FITC (Clone RPA-T4; BD Pharmingen, USA), anti-human CD8-APC-H7 (Clone SK1; BD Pharmingen, USA), anti-human CCR7-PE (Clone REA108; MACS, Germany), and anti-human CD45RA-APC (Clone HI100; BD Pharmingen, USA). Following surface staining, cells were fixed and permeabilized using the Cytofix/Cytoperm kit (BD Biosciences, USA). Intracellular cytokines were detected using anti-human IFN-γ-BV421 (Clone 4S.B3; BD Horizon, USA) and anti-human TNF-α-PE-CF594 (Clone MAb11; BD Horizon, USA). Intracellular staining was performed at 4° C for 40 minutes. Data were acquired using an LSR Fortessa flow cytometer (BD Biosciences) and analyzed with FlowJo™ software version 10.9.0 (BD Life Sciences).

### 2.3 Statistical methods

#### 2.3.1 Hierarchical clustering

We applied hierarchical clustering to identify distinct immune phenotypes based on a comprehensive cytokine profile. A total of 27 cytokines were measured and standardized for analysis. Ward’s method, chosen for its ability to minimize within-cluster variance, was selected for its robust clustering performance. It also achieved the highest agglomerative coefficient (AC = 0.89) compared to other linkage methods, such as single, complete, and average linkage, reinforcing its suitability for our data. The optimal number of clusters was determined to be three, based on an integrated evaluation of total within-cluster sum of squares, average silhouette width, and dendrogram analysis, which collectively indicated the best balance between compactness and separation. To further enhance model relevance and reduce noises, we refined the cytokine dataset by filtering down from 27 to 22 cytokines, retaining only those with significant inter-group differences. This filtering step improved clarity and interpretability of the clustering results. Euclidean distance was used as the metric to calculate dissimilarities between data points.

#### 2.3.2 Survival model development and validation

We analyzed high-dimensional data, including demographic, clinical, and cytokine measurements. To improve the 3-year survival analysis, we performed univariate survival analysis for initial variable selection, retaining only significant variables (**Supplementary Table 2**). Missing data were imputed using random forest; categorical variables were encoded as factors, and continuous variables were standardized. Nested 5-fold cross-validation with 10 replicates optimized model parameters and estimated performance.

An elastic net regularized COX model (Tibshirani, 1996), integrating Lasso (L1) and Ridge (L2) penalties, was employed to identify key predictors of 3-year mortality. Regularization parameters α (alpha, L1-L2 balance) and λ (lambda, penalty strength) were optimized using cross-validation, with an optimal alpha value of 0.1 selected based on the highest mean concordance index (C-index) and minimized variability. We also developed a RSF model (Ishwaran et al., 2008) using nested cross-validation. The outer loop assessed performance; the inner loop optimized key parameters: number of variables tried at each split (mtry), number of trees in the forest (ntree), and minimum size of terminal nodes (nodesize). Final parameters (mtry = 4, ntree = 2000, nodesize = 5) were chosen based on the lowest average out-of-bag (OOB) error.

To compare 36-month survival predictions across the immune phenotypes, we developed a Cox PH model focusing on the EXC group versus the others. A biomarker-based reference model from our previous publication (Wu et al., 2024) was used for benchmarking. Model performances were evaluated using the Concordance index (C-index) on unseen test data, along with the Brier score, calibration plots, and time-dependent area under the curve (AUC).

#### 2.3.3 Statistical analysis

All analyses were performed using R (version 4.4.0) (The R core team, 2024) with relevant packages like tidyverse (Wickham, 2019), MissForest (Stekhoven and Buhlmann, 2012), pheatmap (Kolde, 2019), survminer (Alboukadel Kassambara et al., 2024), survival (Therneau, 2024), glmnet (Friedman et al., 2010), nestedcv (Lewis et al., 2023), randomForestSRC (Ishwaran, 2024), pec (TA, 2023), and timeROC (Blanche et al., 2013). Categorical variables were expressed as frequencies and percentages; continuous variables as means and standard deviations (SD). For group comparisons, we used ANOVA or Kruskal-Wallis tests for continuous variables and χ^2^ or Fisher’s exact tests for categorical variables. Pairwise comparisons utilized the Mann-Whitney U test. Correlations between cytokine levels were analyzed using Pearson’s correlation. Non-linear relationships were modeled using Restricted Cubic Splines (RCS), and Kaplan-Meier (KM) survival curves estimated survival probabilities. Statistical significance was set at p < 0.05.

## 3 Results

### 3.1 Identification and characterization of immune phenotypes

**Figure 1:**
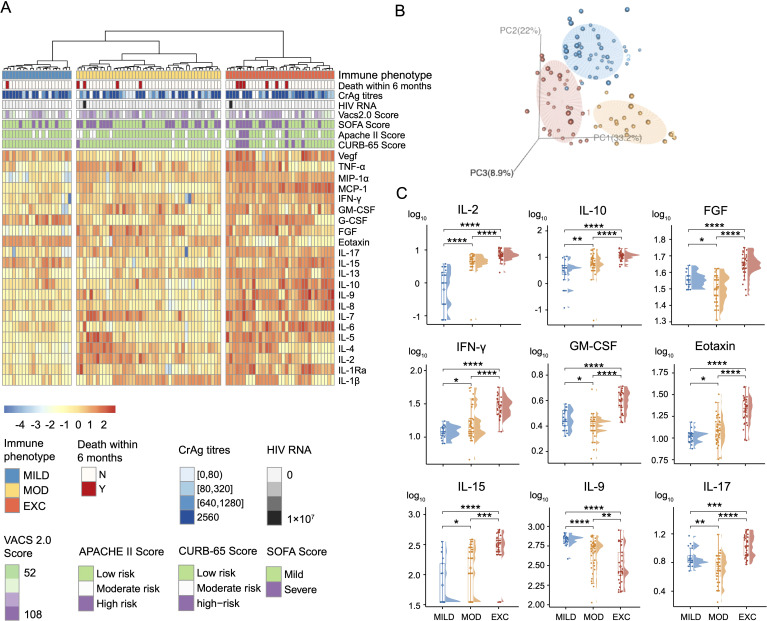
Identifications of three distinct immune phenotype groups based on serum cytokine profiles in HIV-related cryptococcosis cases using hierarchical clustering (N = 98). **(A)** Heatmap illustrating cytokine profiles with severity-related clinical features across the three immune phenotypes (patient distribution: MILD = 21, MOD = 44, EXC = 33). **(B)** 3-dimensional principal component analysis (PCA) plot demonstrating the separation of the three immune phenotypes. **(C)** Comparative analysis of cytokine distributions across the immune phenotype groups. Statistical significance is denoted as: * p < 0.05, ** p < 0.01, *** p < 0.001, and **** p < 0.0001. MILD, Mild immune response; MOD, Moderate immune response; EXC, Excessive inflammatory response; FGF, Fibroblast growth factor; IFN-γ, Interferon-gamma; GM-CSF, Granulocyte-macrophage colony-stimulating factor.

**Figure 2:**
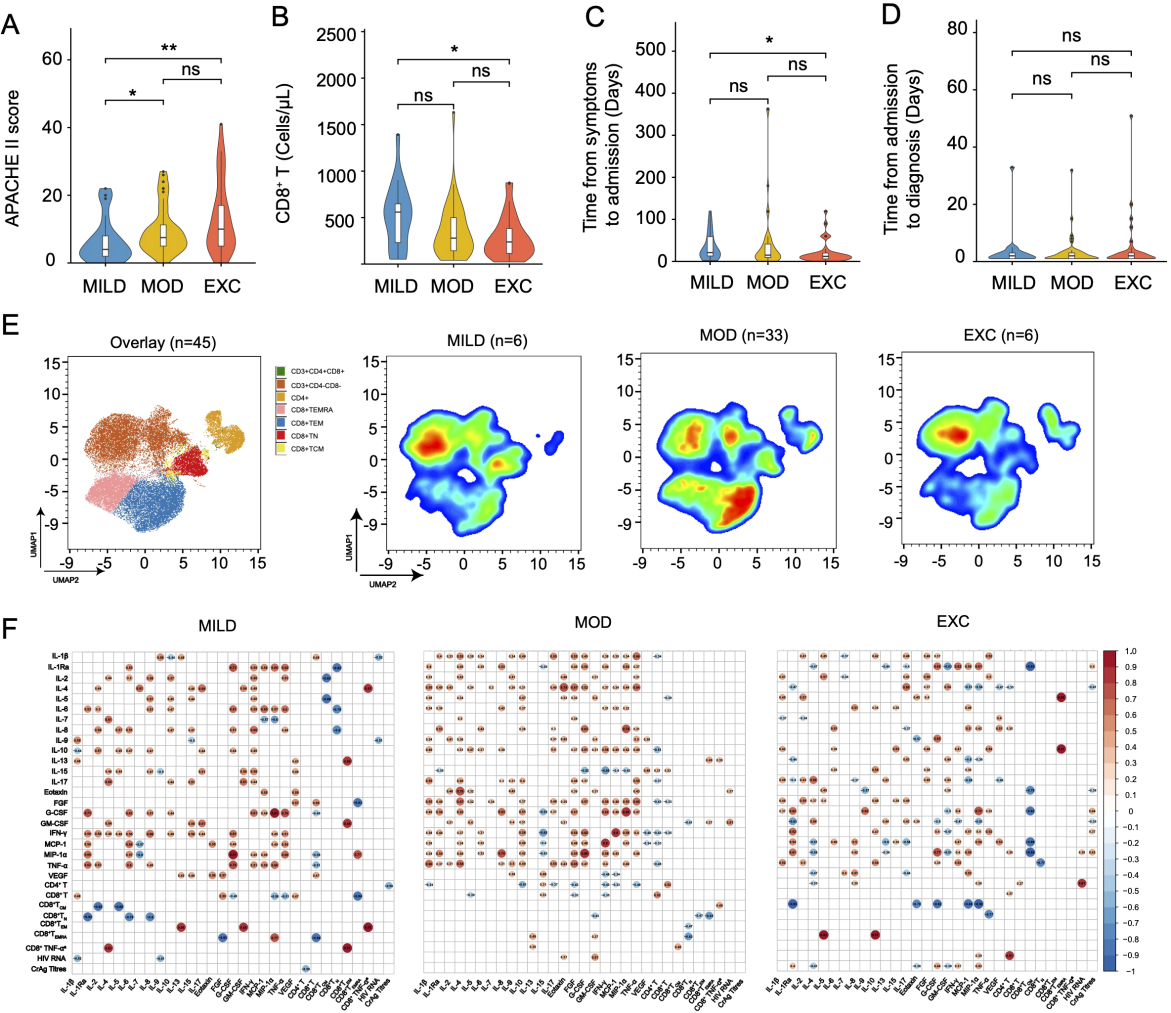
Immune phenotype characterization, high-dimensional flow cytometry analysis, and cytokine correlation in HIV-related cryptococcosis cases across three immune phenotypes (NMILD = 21, NMOD = 44, NEXC = 33). **(A–D)** Violin plots illustrating key clinical features across the three immune phenotypes, with the width of each violin representing the data distribution. **(E)** UMAP plot depicting the separation of the three distinct immune phenotypes (NMILD = 6, NMOD = 33, NEXC = 6) based on high-dimensional flow cytometry data. **(F)** Correlation matrices showing the relationships between immune mediators and pathogen load within each immune phenotype group. Statistically significant positive correlations are represented in red and negative correlations in blue, with color intensity reflecting the correlation strength. Statistical significance for cytokine comparisons between groups is denoted as follows: ns (*p* > 0.05), *p < 0.05, **p < 0.01. UMAP, Uniform Manifold Approximation and Projection; TEM, Effector Memory T Cells; TCM, Central Memory T Cells; TN, Naïve T Cells; TEMRA, Terminally Differentiated Memory T Cells.

Using unsupervised clustering on data from 98 patients with HIV-related cryptococcosis, we identified three distinct immune phenotypes: Mild Immune Response (MILD, n = 21), Moderate Immune Response (MOD, n = 44), and Excessive Inflammatory Response (EXC, n = 33). Cytokine profiles visualized through heatmaps and principal component analysis (**Figures 1A, B**) showed clear separation among the groups. Levels of cytokines such as IL-2, IL-10, IFN-γ, and Eotaxin progressively increased from MILD to MOD to EXC (**Figure 1C**), indicating distinct immune activation patterns correlated with disease severity. Interestingly, IL-9 levels were higher in the MILD group compared to EXC. Demographic characteristics like gender, age, and BMI were similar across groups (**Table 1**). Clinically, the EXC group exhibited a higher prevalence of symptoms such as hearing loss and signs of meningeal irritation. While CD4^+^ T cell counts were uniformly low (<40 cells/μL) across all groups, CD8^+^ T cell counts were significantly lower in the EXC group (287 cells/μL) compared to MILD (515 cells/μL, **Table 1**, **Figure 2B**). Flow cytometry analysis revealed significant differences in immune cell subsets, particularly, CD8^+^ effector memory T cells (T_EM_), across different phenotypes (**Figure 2E**, **Supplementary Figure 3**).

**Table 1:** Clinical and immunological characteristics of HIV-related cryptococcosis cases stratified into three immune phenotype groups: MILD (N = 21), MOD (N = 44), and EXC (N = 33).

	Overall (N=98)	MILD (N=21)	MOD (N=44)	EXC (N=33)	*p*-values
Gender					0.581
Male	13 (13%)	2 (10%)	5 (11%)	6 (18%)	
Female	85 (87%)	19 (90%)	39 (89%)	27 (82%)	
Age (years), Mean (SD)	42.4 (± 13.2)	37.9 (± 12.2)	43.6 (± 12.2)	43.6 (± 14.8)	0.22
BMI (kg/m^2^), Mean (SD)	19.9 (± 3.4)	19.2 (± 2.5)	20.7 (± 4.1)	19.4 (± 2.5)	0.134
Neurological Symptoms	61 (62%)	16 (76%)	21 (48%)	24 (73%)	0.027
Hearing Loss	6 (6%)	1 (5%)	0 (0%)	5 (15%)	0.022
Asymptomatic	23 (23%)	0 (0%)	5 (11%)	18 (55%)	<0.001
Signs of Meningeal Irritation	10 (10%)	0 (0%)	3 (7%)	7 (21%)	0.026
Pachynsis Pleurae	34 (35%)	3 (14%)	21 (48%)	10 (30%)	0.024
CD4 (cells/*μ*L), Mean (SD)	29.7 (± 33.3)	26.1 (± 16.1)	34.0 (± 38.1)	26.1 (± 33.8)	0.518
CD8 (cells/*μ*L), Mean (SD)	363 (± 291)	515 (± 347)	357 (± 293)	287 (± 225)	0.026
HIV-1 RNA (×10^5^ copies/mL), Mean (SD)	5.43 (± 15.8)	2.09 (± 2.01)	6.39 (± 16.6)	6.07 (± 19.1)	0.625
Extrapulmonary dissemination	19 (19%)	3 (14%)	15 (34%)	1 (3%)	0.002
Vacs 2.0 score, Mean (SD)	82.4 (± 10.5)	80.4 (± 10.1)	82.4 (± 10.7)	83.5 (± 10.7)	0.667
SOFA score					0.232
SOFA < 2	59 (60%)	16 (76%)	24 (55%)	19 (58%)	
SOFA ≥ 2	39 (40%)	5 (24%)	20 (45%)	14 (42%)	
APACHE II score					0.071
Low risk [0,15]	4 (4%)	0 (0%)	0 (0%)	4 (12%)	
Moderate risk [16-30]	76 (78%)	18 (86%)	35 (80%)	23 (70%)	
High risk [31,71]	18 (18%)	3 (14%)	9 (20%)	6 (18%)	
CURB-65 severity score					0.006
Low risk [0,1]	8 (8%)	0 (0%)	1 (2%)	7 (21%)	
Moderate risk [2]	85 (87%)	21 (100%)	41 (93%)	23 (70%)	
High risk [3,5]	5 (5%)	0 (0%)	2 (5%)	3 (9%)	
Glasgow score					0.265
Coma [3,8]	20 (20%)	2 (10%)	8 (18%)	10 (30%)	
Moderate [9,11]	3 (3%)	0 (0%)	1 (2%)	2 (6%)	
Mild [12,14]	4 (4%)	1 (5%)	3 (7%)	0 (0%)	
Consciousness [15]	71 (72%)	18 (86%)	32 (73%)	21 (64%)	
Vacs 2.0 score					0.4
[50,83]	39 (40%)	9 (43%)	20 (45%)	10 (30%)	
[83,110]	38 (39%)	6 (29%)	17 (39%)	15 (45%)	
Anti-fungal Treatments					0.004
AmB+5FC ± Flu	64 (65%)	16 (76%)	20 (45%)	28 (85%)	
Flu	17 (17%)	3 (14%)	13 (30%)	1 (3%)	
Others	17 (17%)	2 (10%)	11 (25%)	4 (12%)	

Continuous variables are expressed as mean ± SEM, and categorical variables are presented as counts (percentages). Comparisons across groups were performed using the chi-squared test or Fisher’s exact test for categorical variables and one-way ANOVA or the Kruskal-Wallis test for continuous variables, as appropriate. All severity evaluation scores were calculated based on the maximum values recorded from admission up to 24 hours.

BMI, Body mass index; APACHE II, Acute physiology and chronic health evaluation II; CURB-65, Confusion, urea, respiratory rate, blood pressure, age ≥65; SOFA, Sequential organ failure assessment; VACS, Veterans aging cohort study; AmB, Amphotericin B; FLU, Fluconazole; 5-FC, 5-Fluorocytosine.

### 3.2 Disease severity stratified by immune phenotypes

The EXC phenotype was associated with poorer short-term outcomes and greater disease severity compared to MILD and MOD (**Figure 1A**). Significant differences in CURB-65 severity levels were observed among the three groups (**Table 1**), while SOFA, Glasgow Coma Scale, and VACS 2.0 scores showed no significant associations. Apache II scores were notably higher in the EXC group compared to MILD (**Table 1**, **Figure 2A**). The EXC group had a shorter mean duration from symptom onset to admission (21 days) compared to MILD (35 days), with no significant difference in time to diagnosis (**Figures 2C, D**). Correlation analysis indicated a stronger association between cytokines and cryptococcal antigen (CrAg) titers in the EXC group (**Figure 2F**).

### 3.3 Three-year survival predictive models

**Figure 3:**
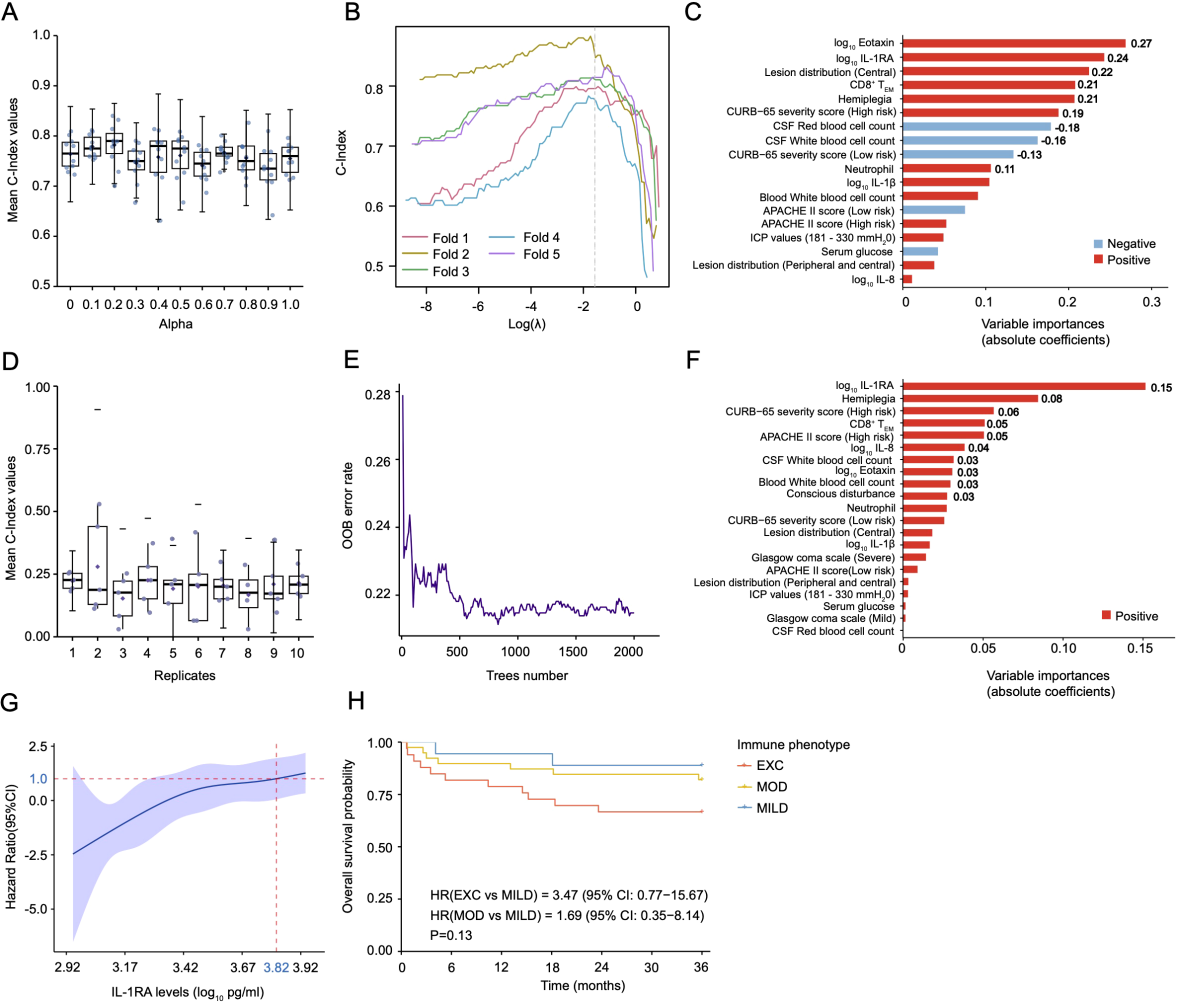
Three-year survival predictive models and variable importance for HIV-related cryptococcosis. **(A–C)** Parameter tuning for alpha and lambda (λ) distributions based on the Concordance Index (C-index) and the variable importance from the penalized Cox model (alpha = 0.1), with features ranked according to their contribution to the model’s predictive performance. **(D–F)** Distribution of the C-index across multiple replicates, out-of-bag (OOB) error rate plot across trees, and variable importance plot from the random survival forest model (trees number: 2000, terminal node size: 5). **(G)** Restricted cubic spline (RCS) curve for the potential biomarker IL1-RA. **(H)** Kaplan-Meier survival curves stratified by the three distinct immune phenotypes(NMILD = 21, NMOD = 44, NEXC = 33) over 36 months, with the p-value from the likelihood ratio test displayed to show the significance of differences between groups. The first 10 variable contribution values were displayed on variable importance plots. C-index, Concordance Index; OOB, Out-of-Bag Error.

To develop a robust 3-year survival prediction model, we constructed a penalized Cox model incorporating immunological and clinical features, optimizing the balance between L1 (Lasso) and L2 (Ridge) penalties. The optimal model (α = 0.1) achieved a mean concordance index (C-index) of 0.78 across 10 replicates (**Figures 3A, B**). As shown in **Figure 3C**, key positive predictors included Eotaxin (coefficient: 0.27) and IL-1RA (coefficient: 0.24), both strong indicators of higher mortality risk. Additional significant contributors were CD8^+^ T_EM_ cells and central lesion distribution. Negative predictors, such as cerebrospinal fluid (CSF) red and white blood cell counts, were associated with reduced mortality risk. In contrast, despite extensive tuning, the RSF model underperformed, with C-index values consistently below 0.5 (**Figures 3D–F**), underscoring the superior predictive capacity of the penalized Cox model in our cohort.

### 3.4 Comparative survival model performance and evaluation

**Figure 4:**
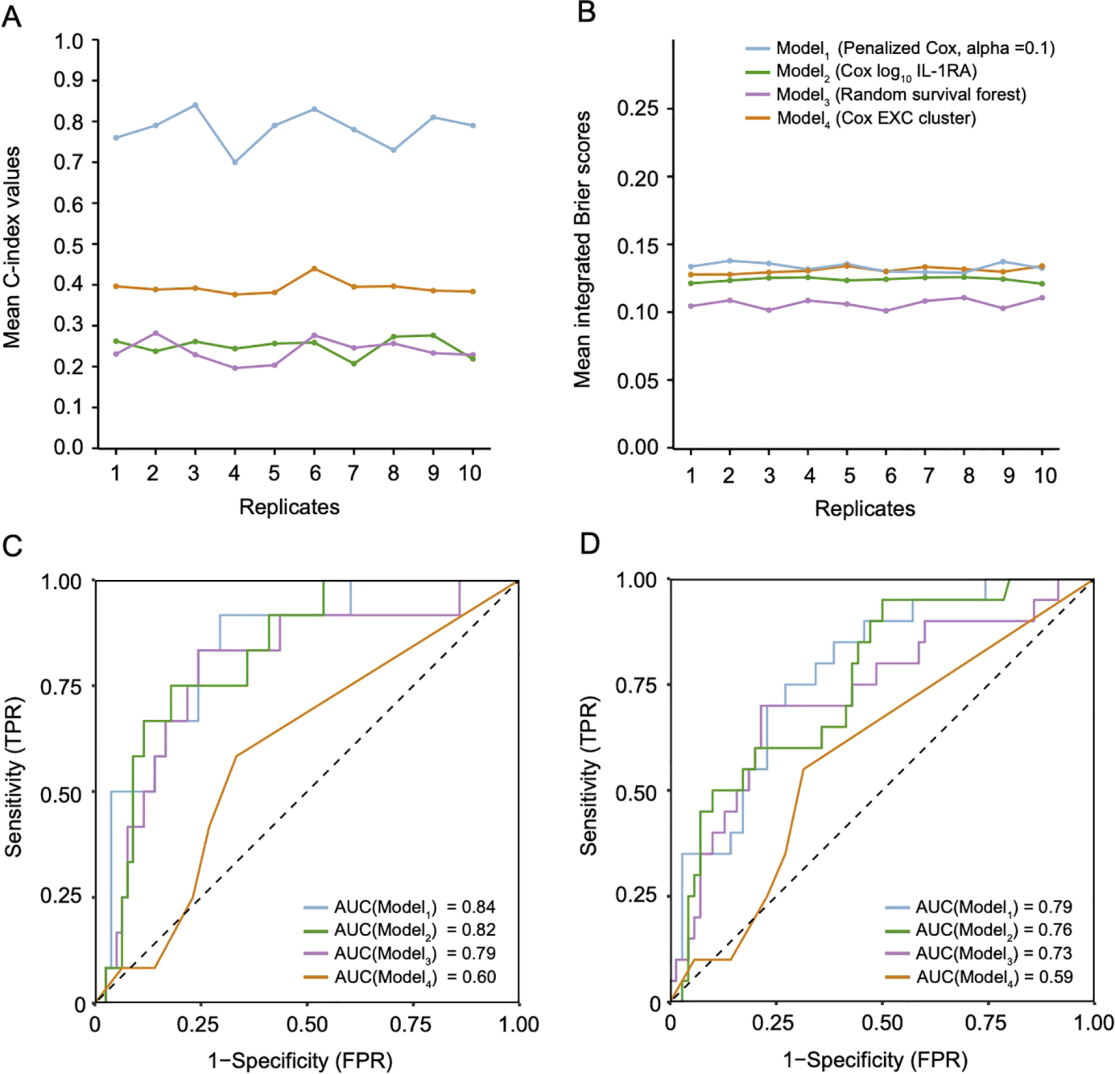
Performance evaluation of survival prediction models for HIV-related cryptococcosis. **(A, B)** Comparative assessments of model performance using the Concordance Index (C-index) and integrated Brier score over 36 months. Lines illustrate the consistency of model performance metrics across multiple replicates. **(C, D)** Time-dependent AUC curves demonstrating variations in model performance at different time points (C: 12 months; D: 36 months). Each line represents the AUC values for a specific model over time, highlighting predictive accuracy at various follow-up durations. All models were evaluated using replicated and nested cross-validation methods to ensure robustness and reliability.

We compared the predictive performance of different survival models, including an optimized Cox model from our previous research. The penalized Cox model (Model 1, α = 0.1) consistently demonstrated the highest mean C-index, indicating exceptional predictive accuracy and consistency (**Figure 4A**). In contrast, the RSF model (Model 3) and the EXCL phenotype-based Cox model (Model 4) exhibited lower and more variable C-index values, reflecting weaker and less reliable performance. The penalized Cox model also had the lowest Brier scores (**Figure 4B**), highlighting its precision in predicting survival probabilities. Calibration plots at 36 months (**Supplementary Figure 6**) showed close alignment between predicted and actual outcomes. Receiver operating characteristic (ROC) analysis at 12 and 36 months (**Figures 4C, D**) further underscored the model’s strong discriminatory power, with AUC values of 0.84 and 0.79, respectively. The IL-1RA-based Cox model (Model 2) performed slightly less well, while the RSF model had limited predictive capacity, indicated by an AUC around 0.60.

## 4 Discussion

This study demonstrates that ML-based methods for risk stratification and survival prediction in HIV-associated cryptococcosis outperform traditional survival analysis in both accuracy and reliability.

As pivotal regulators of immune responses, cytokines are traditionally used to reflect disease progression based on predefined clinical parameters (Shebl et al., 2012), this approach may not capture the full complexity of immune dysregulation in opportunistic infections like cryptococcosis. By employing unsupervised clustering of cytokine data, we uncovered inherent structures and identified natural groupings that were not immediately apparent but correlated with disease stages, progression rates, and treatment responses, which provided deeper insights into the immunopathogenesis of HIV-associated cryptococcosis.

This data-driven subgrouping based on immunological markers has been reported in diseases like COVID-19 (Mueller et al., 2022) and allergic asthma (Muehling et al., 2022), but its application in HIV is limited so far. Our study is the first to identify three distinct immune phenotypes—MILD, MOD, and EXC—in HIV-related cryptococcal cases based on cytokine profiles. The EXC phenotype correlated with the most severe disease presentation, including shorter symptom durations, higher CURB-65 and APACHE II scores, and a threefold increase in 36-month mortality rates compared to the MILD group. Notably, the EXC group had significantly lower CD8^+^ T-cell counts (below 300 cells/μL), while CD4^+^ T cell counts remained uniformly low across all phenotypes. Flow cytometry revealed a significant increase in CD8^+^ T_EM_ cells in the EXCL group compared to the MILD type (**Supplementary Figure 3**). We propose that in advanced HIV infection complicated by cryptococcosis—commonly characterized by CD4^+^ T cell depletion—complex immune phenotypes emerge. One such phenotype is characterized by marked by excessive inflammatory responses and elevated cytokines such as IL-2, IL-10, IFN-γ, and Eotaxin, likely associated with fungal antigen burden (**Figures 1**, **2**). As described previously (Ifergan et al., 2011), CD8^+^ T_EM_ cells demonstrate greater transmigration across the blood-brain barrier compared with non-effector memory CD8^+^ T cells, with selective recruitment further enhanced at the blood-brain barrier endothelium. This phenomenon may underlie the higher prevalence of central nervous system-related symptoms, elevated intracranial pressure and higher CSF/serum albumin ratios observed in our EXC group, ultimately exacerbating clinical outcomes. These findings align with prior studies linking excessive immune activation (cytokine storm) to a dysregulated CNS response in HIV-associated cryptococcosis (Okurut et al., 2020). Notably, the EXC phenotype may indicate a higher-risk subgroup requiring more immediate attention upon admission to slow disease progression and improve survival rates, emphasizing the urgent need for clinicians to recognize these immune phenotypes for better risk assessment and personalized therapeutic interventions. On the other hand, prior studies have also associated elevated CSF IFN-γ levels with enhanced fungal clearance and improved survival in HIV/AIDS patients with cryptococcal co-infection (Jarvis et al., 2012; Jarvis et al., 2015). This apparent contradiction may stem from compartmentalized immune responses and stage-specific variations in host-pathogen interactions (Okafor et al., 2020). Future studies combining paired peripheral and CNS immune profiling with advanced machine learning approaches are needed to elucidate these complex neuroimmune dynamics.

Previous meta-analyses (Li et al., 2024) have reported that the traditional Cox PH model remains the predominant tool for survival analysis in HIV studies, despite its shortcomings in handling overfitting and multicollinearity in high-dimensional data. Addressing the need for fresh analytical approaches, we employed a penalized Cox model incorporating regularization techniques that introduce a penalty term to the loss function. This method effectively addresses small sample sizes and high-dimensional data by shrinking the coefficients of less important variables toward zero, thereby performing variable selection and reducing model complexity (Gui and Li, 2005). To enhance the robustness of our findings, we utilized nested cross-validation with multiple replicates, averaging performance metrics across runs to mitigate the impact of data partitioning peculiarities—a critical consideration when working with limited datasets.

Our optimized penalized Cox model demonstrated superior performance compared to other predictive models, including the previously best-performing traditional Cox PH model based on IL-1RA levels (Wu et al., 2024). While IL-1RA was identified as a potential biomarker for predicting survival in disseminated HIV-associated cryptococcosis, it lacks generalizability across different disease stages. In contrast, the penalized Cox model yielded a mean concordance index of 0.78, a mean Brier score of 0.13, and time-dependent AUC values of 0.84 at 12 months and 0.79 at 36 months. These metrics collectively indicate that our penalized Cox model possesses high accuracy, specificity, sensitivity, and reliability. This model highlighted Eotaxin, IL-1RA, central lesion distribution, CD8^+^ T_EM_, and hemiplegia as key contributors to 36-month mortality risk. These factors partially aligned with findings detected in the EXC phenotype, suggesting their importances in understanding disease progression. We also explored the use of the RSF model in our cohort; however, our findings suggest that it may be unsuitable for small cohorts of patients with complex disorders.

We acknowledge that, despite our efforts to collect data from one of the largest regional HIV care centers in China, recruiting larger cohorts was challenging due to limited patient availability, incomplete data, and variable sample quality. This limitation may impact the statistical power and generalizability of our findings. Additionally, the retrospective design of our study limited the inclusion of CSF cytokines, as lumbar punctures are not routinely performed in all settings. Future multicenter studies with larger cohorts, prospective designs, and CSF-blood paired analyses are needed to validate our findings and strengthen the robustness of our model.

In summary, our study demonstrates the effectiveness of machine learning methods for risk stratification and survival prediction in HIV-related cryptococcosis. Using unsupervised clustering, we identified three distinct immune phenotypes—MILD, MOD, and EXC—providing new insights into immune-related disease severity. The penalized Cox regression model outperformed traditional approaches, highlighting its potential for clinical integration, especially in small and complex cohorts. These findings underscore the need for further research to validate these phenotypes and develop tailored therapeutic strategies, ultimately aiming to improve outcomes for patients with HIV-related opportunistic infections.

## Data Availability

The raw data supporting the conclusions of this article will be made available by the authors, without undue reservation.
