# Supplementary material for: Predictive survival modelings for HIV-related cryptococcosis: comparing machine learning approaches
Source: Front Cell Infect Microbiol. 2025 May 2;15:1542707. doi: 10.3389/fcimb.2025.1542707 (PMC12081334; doi:10.3389/fcimb.2025.1542707)
Supplement: Supplementary file 1 [file DataSheet1.pdf]

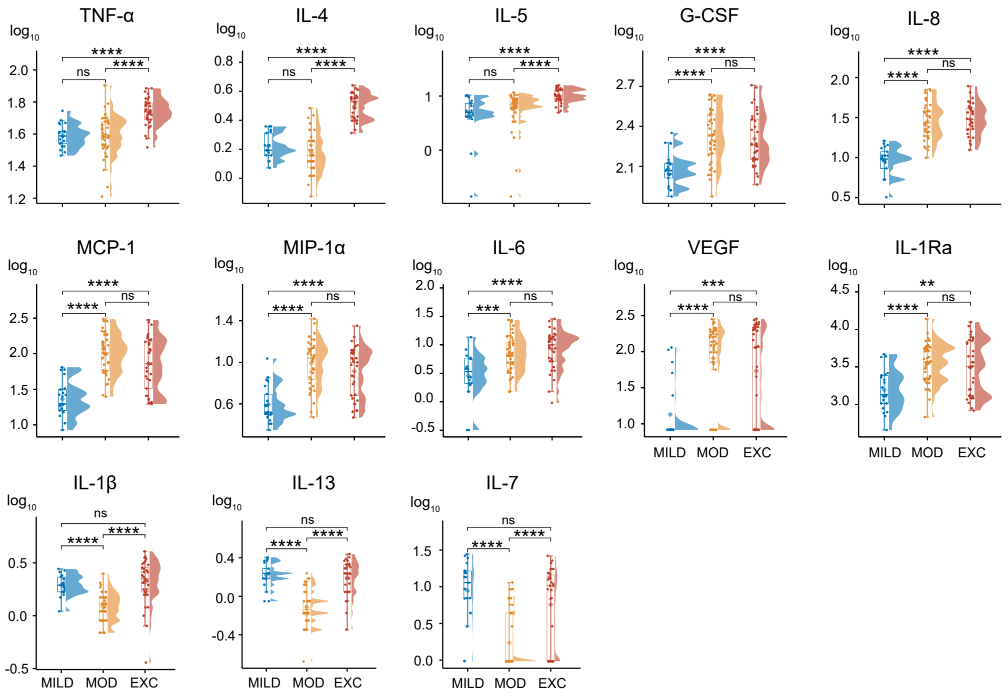

Supplementary Figure 1. Comparative analysis of cytokine distributions across three immune phenotype groups: MILD (N = 21), MOD (N = 44), and EXC (N = 33). Statistical significance between groups is indicated as follows: ns (p > 0.05), * p < 0.05, ** p < 0.01, ***p < 0.001, ****p < 0.0001.
Abbreviations: TNF-α: Tumor Necrosis Factor Alpha; G-CSF: Granulocyte Colony-Stimulating Factor; MCP-1: Monocyte Chemoattractant Protein-1; MIP-1α: Macrophage Inflammatory Protein-1 Alpha; VEGF: Vascular Endothelial Growth Factor; IL-1β: Interleukin-1 Beta.

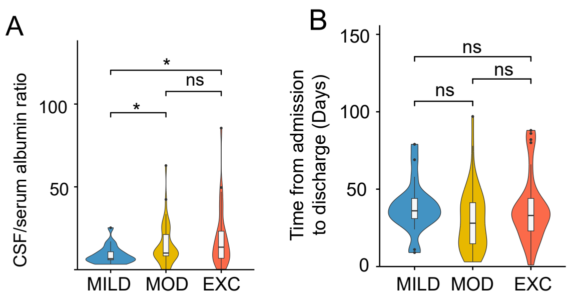

Supplementary Figure 2. Violin plots illustrating key clinical features across the three immune phenotypes (MILD (N = 21), MOD (N = 44), and EXC (N = 33)). The width of each violin represents the data distribution within each group. Statistical significance between groups is indicated as follows: ns (p > 0.05), **p* < 0.05, ** **p** < 0.01, *****p** < 0.001, ******p** < 0.0001.

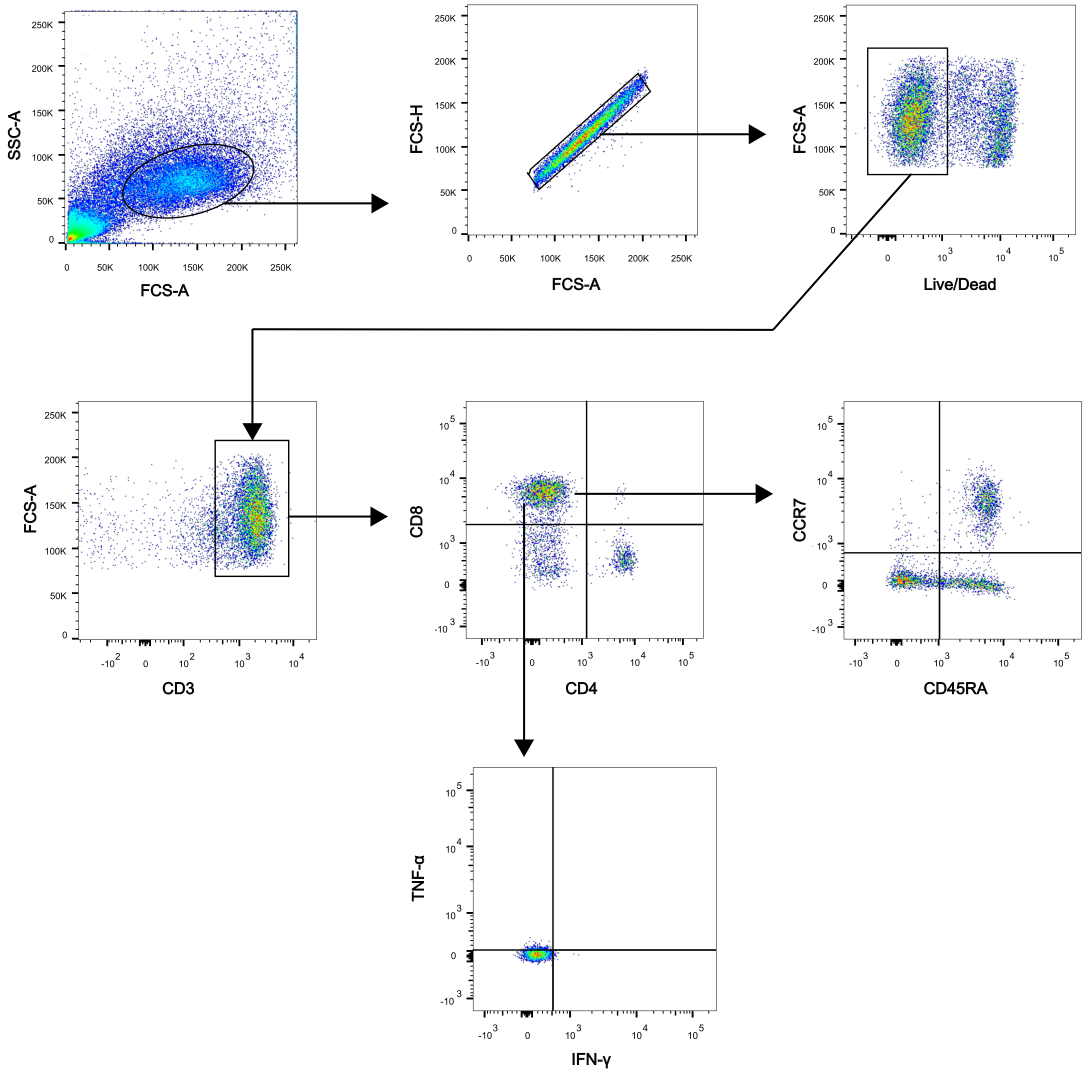

Supplementary Figure 3. Flow cytometry gating strategy.
Sequential gating strategy identifying CD3^{+}, CD4^{+}, and CD8^{+} T cells. CD8^{+} T cell subsets were classified based on CD45RA and CCR7 expression: naive T cells (TN; CD45RA^{+}CCR7^{+}), central memory T cells (TCM; CD45RA-CCR7^{+}), effector memory T cells (TEM; CD45RA-CCR7-), and terminally differentiated effector memory T cells (TEMRA; CD45RA+CCR7-). The production of IFN-γ and TNF-α was analyzed within the CD8^{+} T cell subset.
Abbreviations: CCR7, Chemokine Receptor 7.

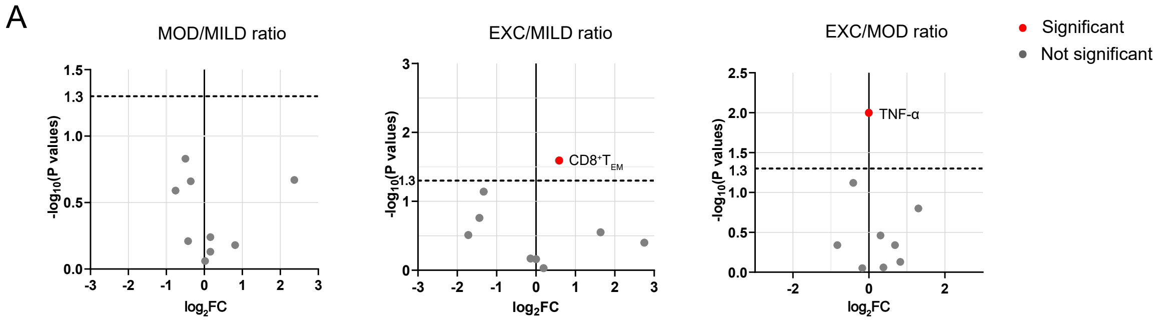

Supplementary Figure 4. Comparative pairwise ratio analysis of high-dimensional flow cytometry-based features across the immune phenotype groups (N_{MILD} = 6, N_{MOD} = 33, N_{EXC} = 6). Each dot represents the ratio of a specific immunological feature between the compared groups, with red dots indicating statistically significant differences. Positive fold-change indicates increase in numerator of ratio, while negative fold-change indicates decreases. Points above the horizontal dashed line (-$\log_{10}$ (0.05) = 1.3) indicate statistically significant differences between groups. The higher a point is on the y-axis, the greater the statistical significance of the difference. Involved indicators as listed: $CD3^+CD4^+CD8^+$, $CD3^+CD4^-CD8^-$, $CD3^+CD4^+CD8^-$（$CD4^+$）, $CD8^+$TCM, $CD8^+$TN, $CD8^+$TEMRA, $CD8^+$TEM, IFN-γ(expressed by C. neoformans-specific $CD8^+$ T cells), TNF-α(expressed by C. neoformans-specific $CD8^+$ T cells))

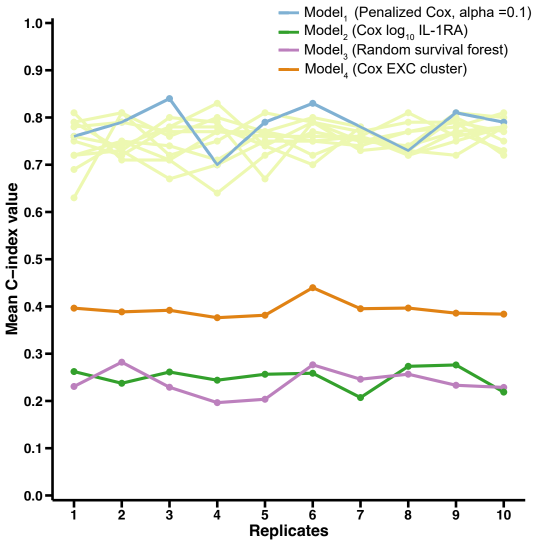

Supplementary Figure 5. Comparative concordance index (C-index) analysis of different model performances over 36 months. Lines illustrate the consistency of model performance metrics across multiple replicates, highlighting the stability and reliability of each model.

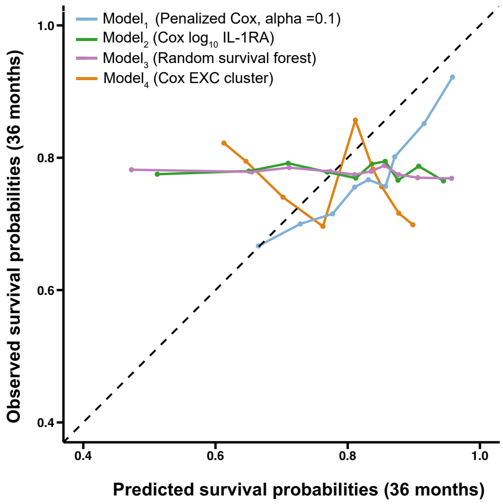

Supplementary Figure 6. Calibration plots comparing the performance of different models over 36 months. The lines represent the alignment between predicted and observed survival probabilities across multiple replicates, indicating model calibration accuracy.
