# Supplementary material for: Predictive survival modelings for HIV-related cryptococcosis: comparing machine learning approaches
Source: Front Cell Infect Microbiol. 2025 May 2;15:1542707. doi: 10.3389/fcimb.2025.1542707 (PMC12081334; doi:10.3389/fcimb.2025.1542707)
Supplement: Supplementary file 2 [file DataSheet2.pdf]

**Clinical features**	**MILD (N=21)**	**MOD (N=44)**	**EXC (N=33)**	**Overall (N=98)**	***P*-values**
**Exposure**					0.478
N	20 (95 %)	43 (98 %)	33 (100 %)	96 (98 %)	
Y	1 (5 %)	1 (2 %)	0 (0 %)	2 (2 %)	
**Comorbidities**					0.766
Diabetes	2 (10 %)	1 (2 %)	0 (0 %)	3 (3 %)	0.129
HBV infection	1 (5 %)	6 (14 %)	2 (6 %)	9 (9 %)	0.382
Malignancies	1 (5 %)	1 (2 %)	3 (9 %)	5 (5 %)	0.403
Chronic kidney disease	1 (5 %)	2 (5 %)	1 (3 %)	4 (4 %)	0.931
**Clinical symptoms**					
Fever	14 (67 %)	31 (70 %)	27 (82 %)	72 (73 %)	0.39
Headache	13 (62 %)	18 (41 %)	10 (30 %)	41 (42 %)	0.071
Nausea	7 (33 %)	11 (25 %)	9 (27 %)	27 (28 %)	0.78
Weight Loss	6 (29 %)	6 (14 %)	7 (21 %)	19 (19 %)	0.344
Sputum	5 (24 %)	14 (32 %)	6 (18 %)	25 (26 %)	0.389
Vomiting	5 (24 %)	13 (30 %)	10 (30 %)	28 (29 %)	0.86
Neck Resistance	5 (24 %)	8 (18 %)	7 (21 %)	20 (20 %)	0.862
Weakness	5 (24 %)	5 (11 %)	7 (21 %)	17 (17 %)	0.358
Conscious Disturbance	4 (19 %)	8 (18 %)	2 (6 %)	14 (14 %)	0.252
Dyspnea	4 (19 %)	6 (14 %)	3 (9 %)	13 (13 %)	0.573
Anorexia	4 (19 %)	3 (7 %)	1 (3 %)	8 (8 %)	0.101
Dizziness	2 (10 %)	6 (14 %)	7 (21 %)	15 (15 %)	0.467

Clinical features	MILD (N=21)	MOD (N=44)	EXC (N=33)	Overall (N=98)	*P*-values
Vision Disorders	2 (10 %)	6 (14 %)	1 (3 %)	9 (9 %)	0.28
Hemiplegia	1 (5 %)	4 (9 %)	2 (6 %)	7 (7 %)	0.783
Chest Distress	1 (5 %)	3 (7 %)	0 (0 %)	4 (4 %)	0.321
Cough	1 (5 %)	3 (7 %)	0 (0 %)	4 (4 %)	0.321
Hemoptysis	1 (5 %)	2 (5 %)	0 (0 %)	3 (3 %)	0.455
Shiver	0 (0 %)	2 (5 %)	4 (12 %)	6 (6 %)	0.163
Chest Pain	0 (0 %)	2 (5 %)	0 (0 %)	2 (2 %)	0.286
**Lesion morphology**					0.759
Nodule shadow	9 (43 %)	14 (32 %)	12 (36 %)	35 (36 %)	
Patchy shadow	5 (24 %)	18 (41 %)	12 (36 %)	35 (36 %)	
Nodule shadow+Patchy shadow	7 (33 %)	12 (27 %)	9 (27 %)	28 (29 %)	
**Lesion location**					
RLL	14 (67 %)	34 (77 %)	25 (76 %)	73 (74 %)	0.643
LLL	13 (62 %)	30 (68 %)	25 (76 %)	68 (69 %)	0.545
RUL	13 (62 %)	24 (55 %)	23 (70 %)	60 (61 %)	0.401
LUL	10 (48 %)	23 (52 %)	23 (70 %)	56 (57 %)	0.189
RML	10 (48 %)	20 (45 %)	20 (61 %)	50 (51 %)	0.395
**Lesion number**					0.621
Multiple	14 (67 %)	29 (66 %)	25 (76 %)	68 (69 %)	
Single	7 (33 %)	15 (34 %)	8 (24 %)	30 (31 %)	

**Clinical features**	**MILD (N=21)**	**MOD (N=44)**	**EXC (N=33)**	**Overall (N=98)**	***P*-values**
**Special signs**					
Cavitations	11 (52 %)	19 (43 %)	17 (52 %)	47 (48 %)	0.693
Intracranial lesion	8 (38 %)	13 (30 %)	15 (45 %)	36 (37 %)	0.281
Enlarged mediastinal lymph node	5 (24 %)	13 (30 %)	8 (24 %)	26 (27 %)	0.83
Pachynsis pleurae	3 (14 %)	21 (48 %)	10 (30 %)	34 (35 %)	0.024
Spiculation Sign	3 (14 %)	3 (7 %)	1 (3 %)	7 (7 %)	0.292
Pleural effusion	2 (10 %)	11 (25 %)	6 (18 %)	19 (19 %)	0.329
Ground glass opacity	2 (10 %)	13 (30 %)	9 (27 %)	24 (24 %)	0.193
Halo sign	1 (5 %)	6 (14 %)	4 (12 %)	11 (11 %)	0.559
Pleural indentation sign	1 (5 %)	3 (7 %)	0 (0 %)	4 (4 %)	0.321
Air bronchogram	1 (5 %)	2 (5 %)	0 (0 %)	3 (3 %)	0.455
Tree in bud pattern	1 (5 %)	1 (2 %)	0 (0 %)	2 (2 %)	0.478
Lobulation Sign	0 (0 %)	1 (2 %)	0 (0 %)	1 (1 %)	0.538
Calcification	0 (0 %)	2 (5 %)	0 (0 %)	2 (2 %)	0.286
**Serum CrAg Titres**	1940 (± 1010)	1520 (± 1160)	1890 (± 977)	1740 (± 1070)	0.239
**WBC ($\times 10^9$/L), Mean (SD)**	7.6 (± 3.8)	4.8 (± 2.7)	5.4 (± 5.8)	5.5 (± 4.3)	0.094
**NE ($\times 10^9$/L), Mean (SD)**	6 (± 3.8)	3.6 (± 2.5)	4.3 (± 5.6)	4.4 (± 4.1)	0.094
**LY ($\times 10^9$/L), Mean (SD)**	0.8 (± 0.4)	0.7 (± 0.5)	0.7 (± 1.2)	0.7 (± 0.8)	0.84
**HB (g/L), Mean (SD)**	119 (± 18.4)	115 (± 21)	112 (± 20.7)	115 (± 20.5)	0.541

Clinical features	MILD (N=21)	MOD (N=44)	EXC (N=33)	Overall (N=98)	*P*-values
**PLT (×10^9/L), Mean (SD)**	200 (± 89.1)	178 (± 75)	204 (± 177)	191 (± 121)	0.598
**TBIL(*μ*mol/L), Mean (SD)**	9.7 (± 4.7)	10.1 (± 4.7)	10.8 (± 4.8)	10.3 (± 4.7)	0.673
**AST (U/L), Mean (SD)**	30.1 (± 26.3)	30.4 (± 25.9)	30.6 (± 25.2)	30.4 (± 25.5)	0.998
**ALT (U/L), Mean (SD)**	23.7 (± 12.2)	26.7 (± 12.9)	30.8 (± 16.3)	27.4 (± 14.1)	0.179
**ALB (g/L), Mean (SD)**	36.3 (± 3.94)	35.7 (± 7.19)	34.0 (± 6.79)	35.3 (± 6.50)	0.361
**Cr (μmol/L), Mean (SD)**	59.9 (± 23.7)	61.1 (± 14.0)	60.0 (± 19.7)	60.5 (± 18.2)	0.955
**BUN (mmol/L), Mean (SD)**	4.9 (± 2.1)	4.7 (± 1.8)	4.8 (± 2.9)	4.8 (± 2.3)	0.943
**LDH (U/L) , Mean (SD)**	268 (± 156)	244 (± 86.7)	288 (± 148)	264 (± 126)	0.317
**CSF white blood cell (×10^9/L), Mean (SD)**	9.75 (± 4.5)	0 (± 0)	13.4 (± 25.6)	12.1 (± 23.2)	0.732
**CSF red blood cell (×10^9/L), Mean (SD)**	254 (± 498)	1.00 (± 1.41)	37.3 (± 108)	62.9 (± 199)	0.117
**CSF total protein (g/L), Mean (SD)**	326 (± 217)	599 (± 445)	588 (± 526)	533 (± 449)	0.054
**ICP (mmH_2O)**					
ICP values <80	1 (5 %)	2 (5 %)	3 (9 %)	6 (6 %)	0.08
ICP values between 80 and 180	5 (24 %)	19 (43 %)	9 (27 %)	33 (34 %)	
ICP values between 181 and 330	7 (33 %)	6 (14 %)	15 (45 %)	28 (29 %)	
ICP values >330	8 (38 %)	10 (23 %)	6 (18 %)	24 (24 %)	
**Co-infections**					0.938

Clinical features	MILD (N=21)	MOD (N=44)	EXC (N=33)	Overall (N=98)	*P*-values
Tuberculosis	2 (10 %)	5 (11 %)	5 (15 %)	12 (12 %)	0.804
CMV	1 (5 %)	3 (7 %)	2 (6 %)	6 (6 %)	0.949
PCP	3 (14 %)	7 (16 %)	5 (15 %)	15 (15 %)	0.985
NTM	4 (19 %)	3 (7 %)	2 (6 %)	9 (9 %)	0.209
Syphilis	3 (14 %)	4 (9 %)	2 (6 %)	9 (9 %)	0.594

Supplementary Table 1. Clinical feature comparisons of HIV-related cryptococcosis cases stratified into three immune phenotype groups: MILD (N = 21), MOD (N = 44), and EXC(N = 33). Continuous variables are expressed as mean ± SEM, and categorical variables are presented as counts (percentages). Comparisons across groups were performed using the chi-squared test or Fisher's exact test for categorical variables and one-way ANOVA or the Kruskal-Wallis test for continuous variables, as appropriate.

Abbreviations: HBV: Hepatitis B Virus; CrAg: Cryptococcal Antigen; WBC: White Blood Cells; NE: Neutrophils; LY: Lymphocytes; HB: Hemoglobin; PLT: Platelets; TBIL: Total Bilirubin; AST: Aspartate Aminotransferase; ALT: Alanine Aminotransferase; ALB: Albumin; Cr: Creatinine; BUN: Blood Urea Nitrogen; LDH: Lactate Dehydrogenase; ICP: Intracranial Pressure; CSF: Cerebrospinal Fluid; CMV: Cytomegalovirus; PCP: Pneumocystis Pneumonia; NTM: Nontuberculous Mycobacteria; LUL: Left Upper Lobe; LLL: Left Lower Lobe; RUL: Right Upper Lobe; RML: Right Middle Lobe; RLL: Right Lower Lobe.

Features	HR	95% CI	P-value
log_{10}Eotaxin	27.69	1.11 – 5.53	0.00
log_{10}IL-1b	14.50	0.21 – 5.14	0.03
log_{10}IL-1RA	13.44	1.07 – 4.12	0.00
APACHE II score (high risk)	11.64	1.33 – 3.58	0.00
CURB-65 (high risk)	8.53	1.18 – 3.11	0.00
log_{10}IL-8	6.12	0.03 – 3.60	0.05
Hemiplegia	4.68	0.44 – 2.65	0.01
Glasgow scale (severe)	3.96	0.49 – 2.26	0.00
Distribution of the lesions (peripheral and central)	3.61	0.05 – 2.51	0.04
Conscious disturbance	2.83	0.03 – 2.05	0.04
Distribution of the lesions (central)	2.46	0.00 – 1.80	0.05
ICP values (181–330 mmH_2O)	2.45	0.02 – 1.77	0.05
Neutrophil	1.12	0.04 – 0.20	0.00
White blood cell count (blood)	1.11	0.03 – 0.19	0.01
$CD8^+$TEM	1.05	0.01 – 0.08	0.00
Red blood cell count (CSF)	0.99	–0.02 – 0.00	0.01
White blood cell count (CSF)	0.90	–0.19 – –0.02	0.01
Glasgow scale (mild)	0.35	–1.94 – –0.17	0.02
Serum glucose	0.34	–2.03 – –0.11	0.03
APACHE II score (low risk)	0.27	–2.20 – –0.44	0.00
CURB-65(low risk)	0.18	–2.64 – –0.79	0.00

Supplementary Table 2. Variables identified as significantly different from univariate 36-month survival analysis. Each variable is listed with its corresponding statistical significance, highlighting factors associated with survival outcomes over the 36-month follow-up period.

Abbreviations: HR, Hazard Ratio; CI: Confidence Interval.

Alpha Value	Mean C-Index	Standard Deviation	Minimum C-Index	Maximum C-Index	C-Index Range
0.0	0.76	0.03	0.72	0.81	0.09
0.1	0.78	0.02	0.75	0.81	0.06
0.2	0.78	0.04	0.70	0.84	0.14
0.3	0.75	0.04	0.67	0.79	0.12
0.4	0.76	0.06	0.63	0.81	0.18
0.5	0.76	0.04	0.67	0.81	0.14
0.6	0.74	0.03	0.69	0.78	0.09
0.7	0.77	0.02	0.73	0.80	0.07
0.8	0.76	0.04	0.70	0.83	0.13
0.9	0.73	0.05	0.64	0.81	0.17
1.0	0.76	0.03	0.71	0.80	0.09

Supplementary Table 3. Summary statistics of the concordance Index (C-index) for different alpha parameter tuning in the penalized Cox model. All models were evaluated using nested cross-validation, and all values are calculated based on 10 replicates, providing a robust assessment of model performance across varying alpha parameters.
